# Investigation of the Effect of Enamel Matrix Protein, Platelet-Rich Fibrin, and Bone Graft on New Bone Formation in Guided Tissue Regeneration in Rat Calvarium

**DOI:** 10.3390/medicina61101795

**Published:** 2025-10-04

**Authors:** Tuğçe Dönmezer, Tuba Talo Yildirim, Serkan Dündar, Alihan Bozoğlan, İbrahim Hanifi Özercan

**Affiliations:** 1 Department of Periodontology, Faculty of Dentistry, Tekirdağ Namık Kemal University, 59000 Tekirdağ, Turkey; 2 Department of Periodontology, Faculty of Dentistry, Fırat University, 23000 Elazığ, Turkey; 3 Department of Medical Pathology, Faculty of Medicine, Fırat University, 23000 Elazığ, Turkey

**Keywords:** enamel matrix protein, guided tissue regeneration, platelet-rich fibrin, titanium, membrane, Xenograft, OPG, RANKL, model antitumor assays

## Abstract

*Background and Objective*: The aim of this study was to evaluate the effects of enamel matrix protein, platelet-rich fibrin (PRF), and bone graft on new bone formation beyond the skeletal system by creating calvarial bone defects in rats. The effects were assessed using histopathological and immunohistochemical analyses. *Materials and Methods*: In this study, calvarial bone defects were created in male Sprague Dawley rats weighing 500–550 g. The animals were randomly divided into seven groups: Control (*n* = 13), Emdogain (EMD, *n* = 13), Emdogain + Bone Graft (EMD + BG, *n* = 13), Platelet-Rich Fibrin (PRF, *n* = 13), PRF + Bone Graft (PRF + BG, *n* = 13), Bone Graft (BG, *n* = 13), and PRF + Emdogain + Bone Graft (PRF + EMD + BG, *n* = 13). An additional group of 36 rats was used for PRF preparation. Titanium domes were placed on the calvarial bone defects, and the animals were sacrificed after three months. Bone samples were evaluated histopathologically for new bone formation, numbers of osteoblasts and osteoclasts, angiogenesis, and fibrosis. Immunohistochemical analysis of bone formation was performed using OPG and RANKL staining kits. Data were analyzed statistically. *Results*: The PRF group showed a significantly higher level of moderate new bone formation compared with the PRF + BG, EMD + BG, and PRF + EMD + BG groups (*p* ≤ 0.05). No significant differences were observed among the groups in terms of fibrosis or angiogenesis (*p* > 0.05). Similarly, OPG and RANKL levels, as well as the OPG/RANKL ratio, did not differ significantly between groups (*p* > 0.05). *Conclusions*: Based on the findings of this study, the combined use of Emdogain, PRF, and bone graft appears to have beneficial effects on enhancing bone formation in calvarial defects.

## 1. Introduction

Guided tissue regeneration (GTR) is based on the principle of preventing undesirable tissue from migrating into the defect area through the use of barrier membranes. It is widely considered a promising method for managing the destruction of periodontal tissue caused by periodontitis [1]. GTR has been applied in the treatment of various periodontal syndromes, including intrabony defects, furcation involvement, and localized gingival recession [2]. Compared to simple repair, regenerative periodontal therapy provides superior healing through the reconstruction of periodontal tissues. Previous studies have demonstrated that regenerative periodontal therapy provides greater clinical improvement than conventional therapies. Successful regenerative periodontal therapy requires biomaterials that facilitate regeneration of the periodontal ligament, along with the formation of new attachment, cementum, and alveolar bone [3]. GTR involves the use of barrier membranes in combination with autologous bone and bone marrow, allografts, xenografts, and various bone graft materials, as well as bioactive growth factors such as bone morphogenetic proteins (BMPs) and enamel matrix proteins [4].

Enamel matrix proteins have been used as therapeutic agents in GTR since 1997 under the commercial name Emdogain (Straumann AG, Basel, Switzerland), a synthetic gel containing enamel matrix derivative (EMD) harvested from developing six-month-old porcine tooth buds. Emdogain is approved by the United States Food and Drug Administration. The EMD consists of enamel matrix-derived proteins, water, and a carrier composed of propylene glycol alginate [5]. The proteins in the enamel matrix include amelogenin, enamelin (ENAM), ameloblastin (AMBN, also known as amelin or sheathlin), amelotin (AMTN), odontogenic ameloblast-associated (ODAM/APin), transforming growth factor beta (TGF-β), BMPs, and various proteinases [6]. The addition of these proteins to intrabony defects can enhance periodontal regeneration in dental treatments [7]. EMD has been reported to contain bone sialoprotein-like substances that are known to promote angiogenesis through their receptors [8]. For intrabony defects, treatment with flap surgery combined with EMD has been shown to result in significantly greater clinical attachment gains compared to flap surgery alone, positively influencing the prognosis of periodontal therapy [9].

One study demonstrated that 3D scaffolds produced from the patient’s peripheral blood can be utilized in GTR [4]. Following this approach, platelet-rich fibrin (PRF), a matrix derived from platelet concentrates without the use of anticoagulants, is often employed in GTR. PRF is an advanced formulation of platelet-rich plasma (PRP) [10]. Choukroun’s PRF is a biomaterial rich in both leukocytes and platelets [11]. When used as a membrane, PRF not only protects the surgical site postoperatively but also accelerates the adaptation and remodeling of the biomaterial [12]. In its gel form, PRF enhances wound healing as well as bone growth and maturation, reduces wound permeability, ensures hemostasis, and improves the handling characteristics of graft materials. Numerous clinical studies have recommended the combined use of bone grafts and PRF to increase bone density [13].

Accelerating bone regeneration and achieving more stable bone remodeling are crucial factors in the treatment of periodontal defects. The rat calvarial defect model, widely recognized for its standardized creation of critical-size defects, low morbidity, and well-characterized healing patterns, serves as a reliable preclinical platform for evaluating bone regenerative approaches [14]. In this study, male Sprague Dawley rats aged 6–12 months were selected to minimize potential hormonal variations and ensure consistent bone physiology and regenerative responses. To maintain the regenerative space and prevent soft tissue collapse, titanium domes were placed over the defects, in line with the principles of guided bone and tissue regeneration. Within this framework, the present study aimed to investigate the effects of Emdogain (EMD), platelet-rich fibrin (PRF) and bone xenografts in combination with guided tissue regeneration (GTR) on bone formation, using histopathological and immunohistochemical analyses. The findings may provide valuable insights into which biomaterial promotes faster and more effective bone regeneration, thereby informing future clinical treatment strategies.

## 2. Materials and Methods

This study was approved by the Animal Experiments Ethics Committee of Fırat University (Elazığ, Turkey; project number: 97132852/604.01.02; approval number: 19.08; approval date: 28 April 2019); the committee also approved the withdrawal of one of the initially listed researchers (Assoc. Prof. Dr. Gökhan Artaş) on 31 May 2019, and the study continued with the revised research team. All animals were obtained from the Experimental Research Center of Fırat University (Elazığ, Turkey), where the experimental procedures were also conducted. Histological and immunohistochemical sectioning and analyses were performed in the Department of Pathology, Faculty of Medicine, Fırat University (Elazığ, Turkey).

### 2.1. Animals and Study Design

**Figure 1:**
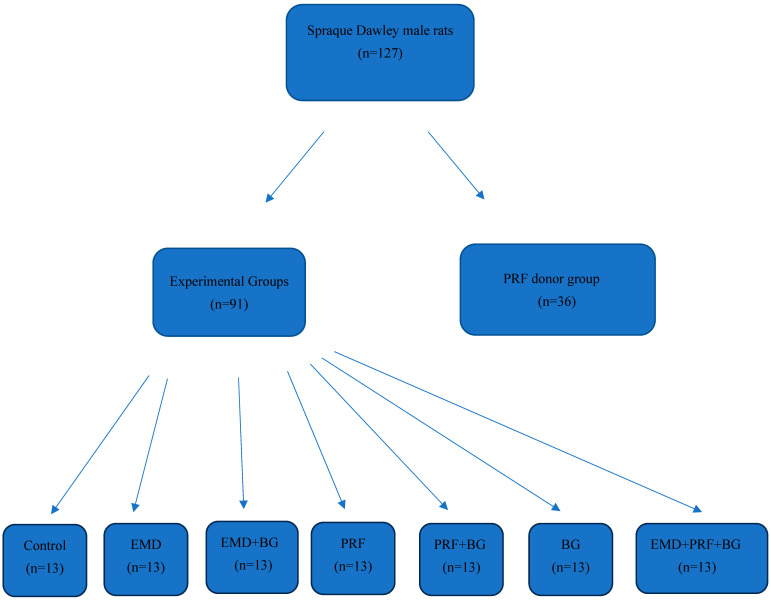
Experimental setup of the animal study.

This experimental study involved 127 male Sprague Dawley rats weighing 500–550 g and aged 6–12 months: 91 were used in the experimental groups, and 36 were used to prepare the PRF. Sample size estimation was performed using G*Power software (Version 3.1.9.7) [15]. The analysis was conducted with α = 0.05, power (1-β) = 0.95, and degrees of freedom = 12. Based on effect sizes reported in the literature, Cohen’s w was set at 0.55. Accordingly, the required minimum sample size was calculated as 86. In line with this estimation, a total of 91 animals were allocated to the experimental groups to ensure sufficient statistical power (Figure 1).

The rats did not receive any additional nutritional supplements before, during, or after the experiment. The rats were housed in specially ventilated cages, ensuring that they were not deprived of any environmental needs, and had ad libitum access to food and water.

The PRF was prepared from blood collected from the left ventricle of 36 rats [16]. For all rats in the experimental groups, nine cavities measuring 1 mm in diameter and 1.5 mm in depth were created on the calvarial bone. However, the subsequent treatment varied between the experimental groups as follows:

- Control Group (*n* = 13): Titanium domes were placed over the decorticated cavities, leaving their interior empty.
- Emdogain Group (EMD, *n* = 13): Titanium domes filled with Emdogain were placed over the decorticated cavities.
- Emdogain and Bone Graft Group (EMD + BG, *n* = 13): Titanium domes filled with a mixture of bone xenograft and Emdogain were placed over the decorticated cavities.
- PRF Group (PRF, *n* = 13): Titanium domes filled with PRF were placed over the decorticated cavities.
- PRF and Bone Graft Group (PRF + BG, *n* = 13): Titanium domes filled with a mixture of bone xenograft and PRF derived from the rats’ blood were placed over the decorticated cavities.
- Bone Graft Group (BG, *n* = 13): Titanium domes filled with bone xenograft were placed over the decorticated cavities.
- Emdogain, PRF, and Bone Graft Group (EMD + PRF + BG, *n* = 13): Titanium domes filled with a mixture of Emdogain, PRF, and bone xenograft were placed over the decorticated cavities.

Following the experimental procedures, the rats were maintained on a normal diet for three months. At the end of this period, the rats were sacrificed, and their calvarial bones were harvested for histopathological and immunohistochemical examination [17].

Following the procedures, the animals were maintained on a normal diet for 3 months. At the end of this period, the subjects were sacrificed, and calvarial bones were harvested for histopathological and immunohistochemical examination. Only a single 12-week endpoint was assessed. Quantitative 3D micro-CT analysis was not performed due to budgetary and technical constraints; histopathological and immunohistochemical analyses were used instead [17].

### 2.2. Surgical Method

All surgical procedures were performed under sterile conditions and general anesthesia. General anesthetics (10 mg/kg Xylazine and 40 mg/kg ketamine) were administered intramuscularly using an appropriate syringe. A single 0.5 cc dose of 4% articaine containing 0.006 mg/mL epinephrine was injected subcutaneously to control bleeding in the surgical area. Next, the surgical site was shaved and disinfected with povidone-iodine before the procedure. Then, a midline incision was made along the calvarial bones using a No. 15 scalpel blade. Next, nine decortication defects, each measuring 1 mm in diameter and 1.5 mm in depth, were created on the parietal bone. To prevent overheating during the drilling process, the surgical area was irrigated continuously with sterile saline solution [17]. Then, the titanium domes were placed.

**Figure 2:**
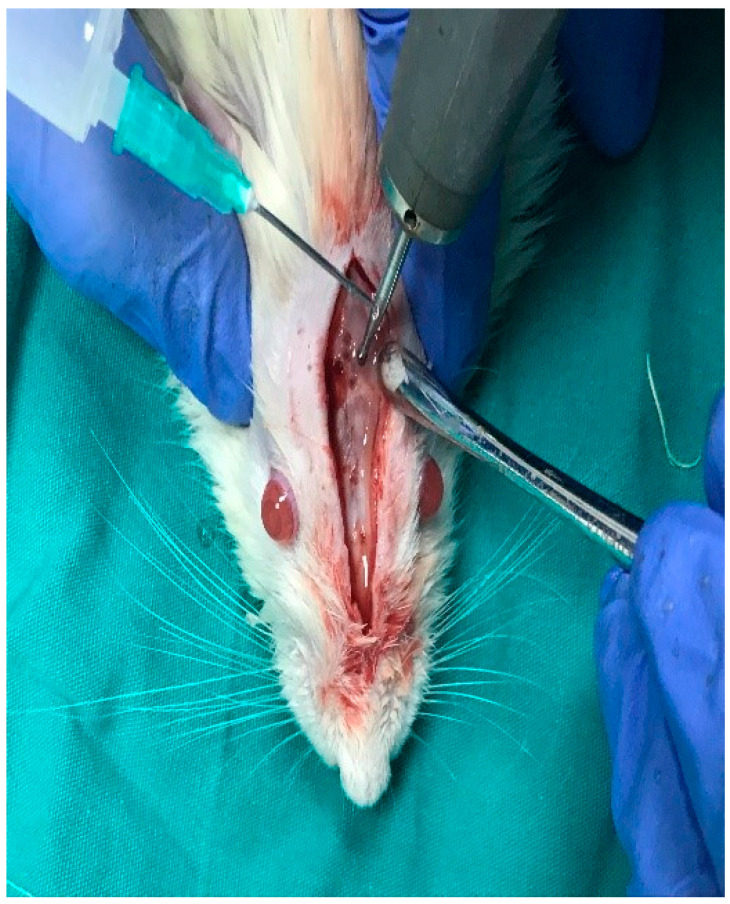
Standardized 9 mm decortication cavities were created in the calvaria of rats under sterile conditions.

**Figure 3:**
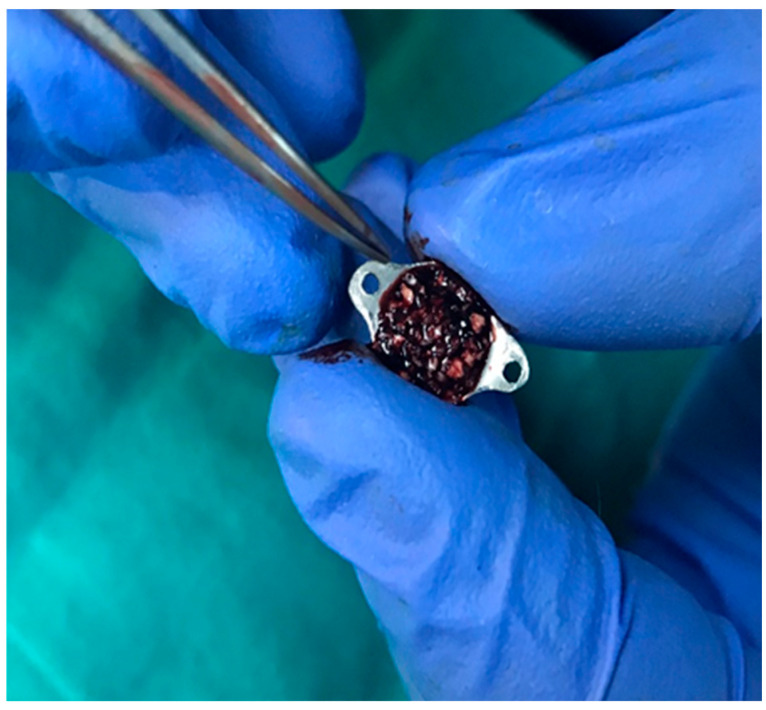
Titanium domes filled with graft materials.

**Figure 4:**
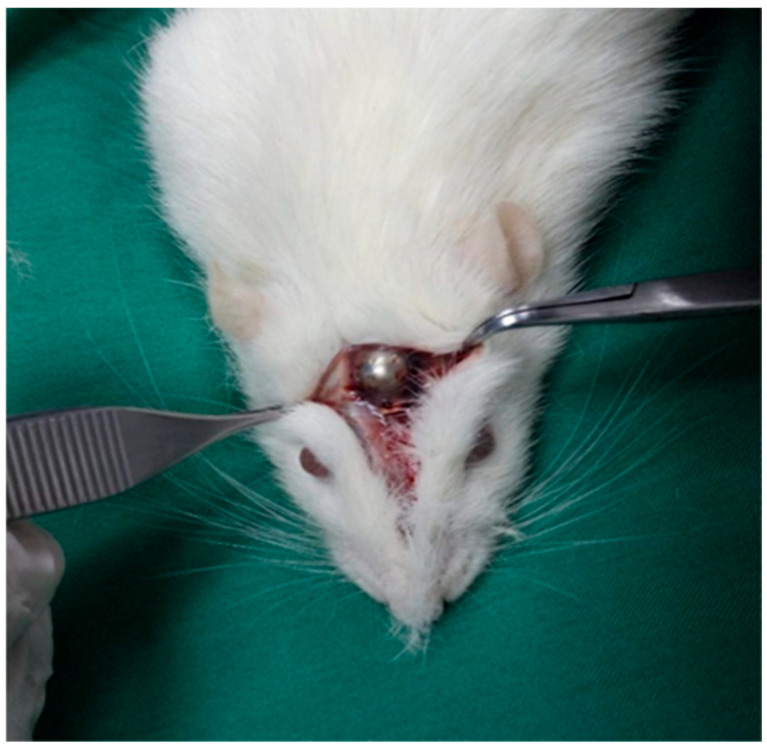
Titanium domes filled with graft materials were placed onto the calvarial bones using PeriAcryl®90, GluStitch Inc., Saint Paul, MN, USA.

This study utilized titanium domes measuring 6 mm in diameter and 3 mm in height (Figure 2). The domes were filled with PRF, Emdogain (EMD; Straumann, Basel, Switzerland), and/or 7 cc (2 + 5 cc) of bovine-derived xenograft (BG; Bonefill; Bionnovation, Ankara, Turkey). The titanium domes were fixed onto the calvarium using a tissue adhesive, N-butyl-2-cyanoacrylate (PeriAcryl^®^90, GluStitch Inc., Saint Paul, MN, USA), which prevented soft tissue invasion into the titanium barriers, thereby facilitating GTR beyond the skeletal system to promote bone formation (Figure 3 and Figure 4). The skin and subcutaneous tissues were sutured with 3/0 polyglactin 910 (Vicryl; Ethicon, Cincinnati, OH, USA). Following all surgical procedures, the rats were administered antibiotics (40 mg/kg cephalosporin) and analgesics (0.1 mg/kg tramadol hydrochloride) intramuscularly for three days to prevent infection and manage pain [17].

### 2.3. PRF Preparation

**Figure 5:**
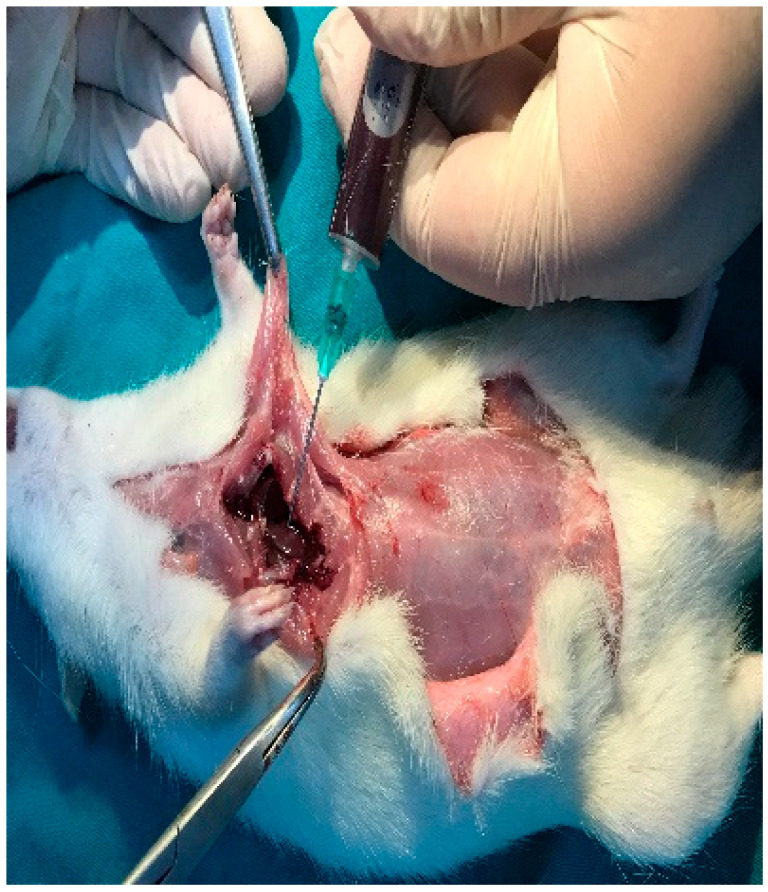
Blood was collected from rats using the open-heart method.

**Figure 6:**
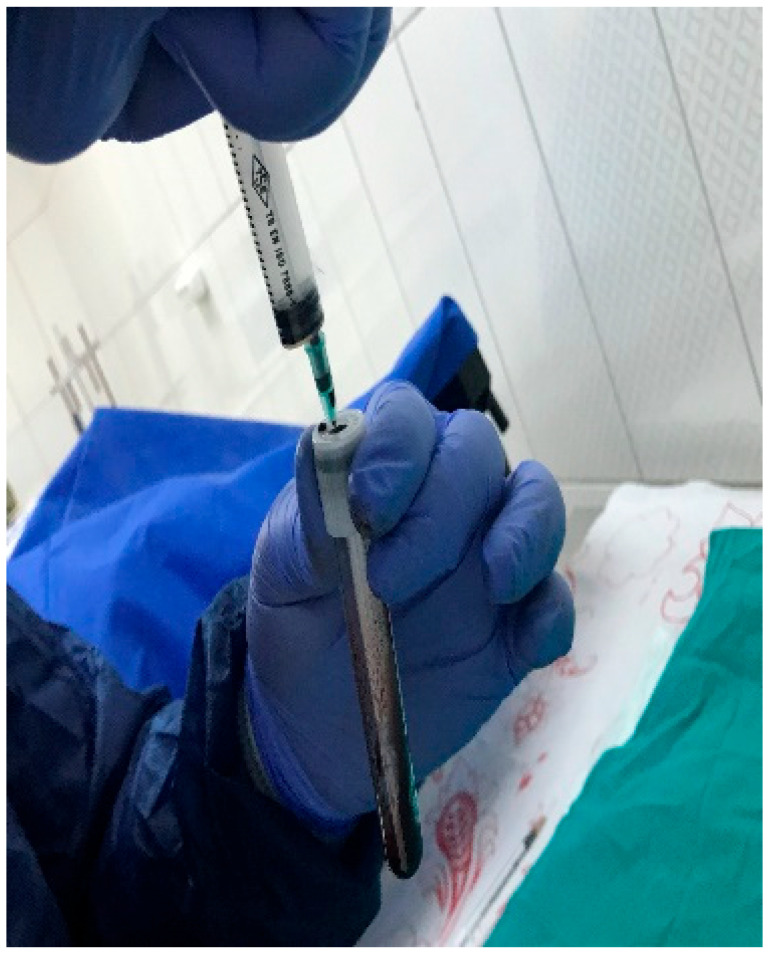
Blood collected from rats was placed into PRF tubes.

**Figure 7:**
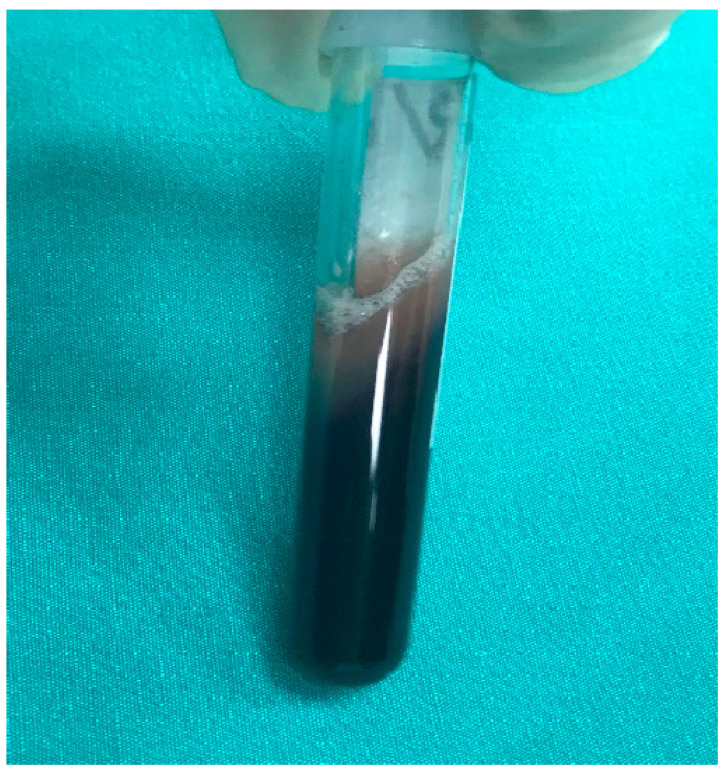
The collected blood was centrifuged to obtain PRF.

All surgical procedures were performed under sterile conditions and general anesthesia. General anesthetics (3 mg/kg Xylazine and 60 mg/kg ketamine) were administered intramuscularly using an appropriate syringe. Next, approximately 5 mL of blood was aspirated from the left ventricle of the heart into anticoagulant-free tubes. Then, the blood samples were centrifuged at 3000 rpm for 10 min (Nüve NF 200 Centrifuge, Elazığ, Turkey) (Figure 5, Figure 6 and Figure 7). Finally, PRF was isolated from the centrifuged tubes, cut into small pieces using sterile scissors, and placed into titanium domes [16].

### 2.4. Calvarial Bone Collection and Preparation

**Figure 8:**
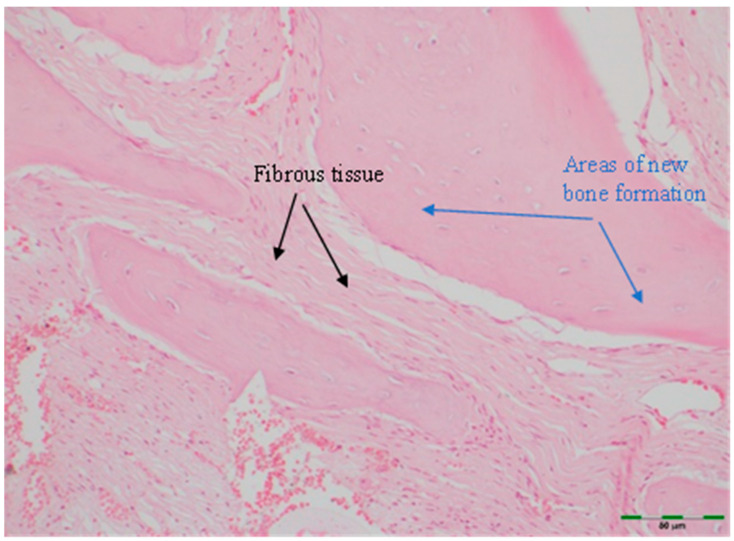
Histological appearance of mild new bone formation and dense fibrous tissue in the control group (Hematoxylin and Eosin staining, 20× magnification). Arrows indicate areas of new bone formation (blue) and fibrous tissue (black). Scale bar: 50 µm.

**Figure 9:**
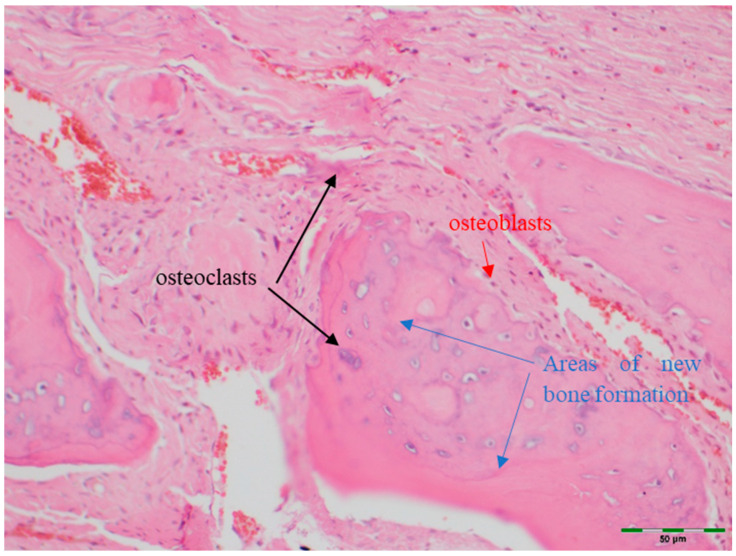
Histological appearance of the EMD group showing moderate new bone formation with high level osteoblasts and osteoclasts (Hematoxylin and Eosin staining, 20× magnification). Arrows indicate areas of new bone formation (blue),osteoblasts (red) and osteoclasts (black). Scale bar: 50 µm.

**Figure 10:**
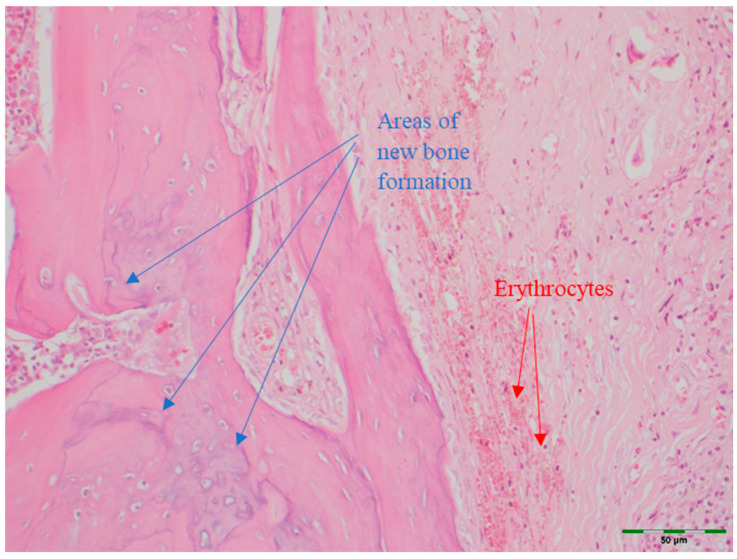
Histological appearance of the PRF + BG group showing high level of new bone formation and prominent areas of angiogenesis (Hematoxylin and Eosin staining, 20× magnification). Arrows indicate regions of new bone formation (blue) and angiogenesis (red). Scale bar: 50 µm.

**Figure 11:**
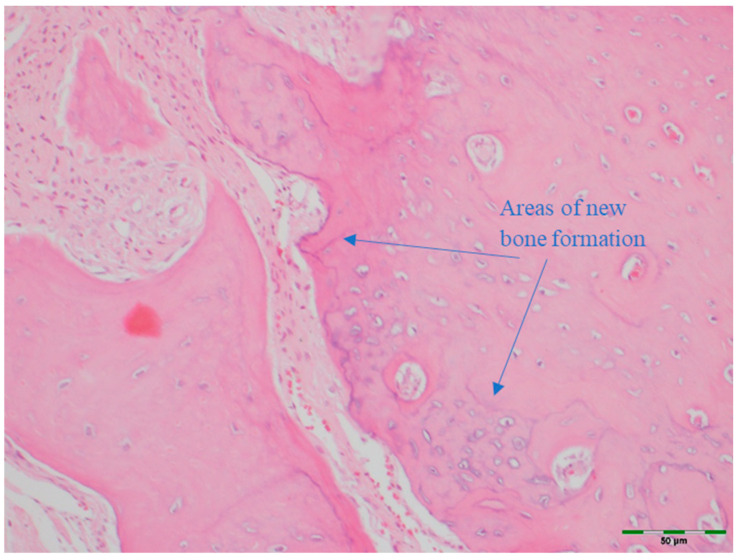
Histological appearance of the PRF group showing moderate new bone formation (Hematoxylin and Eosin staining, 20× magnification). Arrows indicate areas of new bone formation (blue). Scale bar: 50 µm.

**Figure 12:**
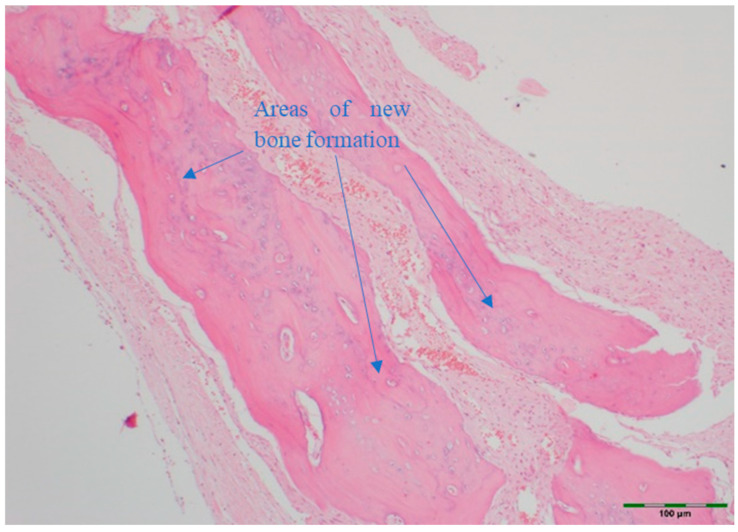
Histological appearance of the EMD + PRF + BG group showing a high level of new bone formation (Hematoxylin and Eosin staining, 20× magnification). Arrows indicate areas of new bone formation (blue). Scale bar: 100 µm.

The rats were sacrificed at the end of a 12-week healing period. After sacrifice, the titanium domes were removed, and the calvarial bones containing newly formed bone tissue were collected and preserved in 10% formalin under appropriate conditions for histopathological and immunohistochemical examination [17]. The preparation of the histological sections and the histological and immunohistochemical examinations of the bone tissues were performed in the Department of Pathology, Faculty of Medicine, Fırat University. First, the calvarial bones samples were fixed in 10% formalin at room temperature for 72 h. Decalcification was performed in 10% formic acid at room temperature, with the solution replaced every 2 days, and was completed within 1 week. Following decalcification, the samples were thoroughly washed under running water for 12 h to remove residual acid before further processing [15,17]. Next, they were decalcified in 10% formic acid. Then, they were washed for a specific period in running water to remove residual formic acid. Following routine processing, the tissue samples were embedded in paraffin blocks and sliced into 5 µm thick sagittal sections using a microtome (GENBİYO Technology Operations Laboratory, Elazığ, Turkey). Next, the sections were deparaffinized at 65 °C for one hour, incubated in xylene and alcohol, washed in water, and then stained with hematoxylin and eosin (H&E). Decalcified bone sections (5 µm) were deparaffinized at 65 °C for 1 h, followed by incubation in xylene and graded alcohols for complete deparaffinization and rehydration. Sections were washed in running water and stained with hematoxylin for 5 min at room temperature, differentiated in 1% acid alcohol, blued in 0.2% ammonia water, and counterstained with eosin for 2 min at room temperature. Finally, sections were dehydrated, cleared in xylene, and mounted with coverslips. This protocol follows standard procedures as described previously (Table 1) [15]. Finally, the H&E-stained sections were examined under a light microscope (BX51; Olympus, Tokyo, Japan) to assess the following parameters:

(a) Amount of newly formed bone: New bone formation was histologically evaluated by identifying and scoring the presence of layered bone tissue. Unlike mature bone, lamellar bone tissue exhibits trabeculation and bone marrow. Bone formation was scored as follows (Figure 8, Figure 9, Figure 10, Figure 11 and Figure 12) [18]:

0 = none.

1 = mild.

2 = moderate.

3 = extensive.

(b) Osteoblasts: The density of osteoblasts within the total defect area was visually identified and scored as follows (Figure 9) [18]:

0 = none.

1 = mild.

2 = dense.

(c) Osteoclasts: The density of osteoclasts within the total defect area was visually identified and scored as follows (Figure 9) [18]:

0 = none.

1 = mild.

2 = dense.

(d) Fibrosis: Fibrosis was visually identified and scored as follows (Figure 8) [19]:

0 = absent.

1 = mild.

2 = moderate.

3 = severe.

(e) Angiogenesis: Angiogenesis was visually identified and scored as follows (Figure 10) [20]:

0 = none.

1 = mild.

2 = moderate.

3 = extensive.

### 2.5. Immunohistochemical Examination

OPG and RANKL were analyzed to evaluate bone remodeling activity. An avidin–biotin–peroxidase complex procedure was applied to determine the immunoreactivities of TNF superfamily member 11 (TNFSF11/RANKL) and TNF receptor superfamily member 11b (TNFRSF11B/OPG) in the calvarial bone tissues of the rats. Tissue sections, 5 μm in thickness, were prepared on poly-L-lysine-coated slides, deparaffinized, and rehydrated. After deparaffinization and rehydration, sections were treated with 3% hydrogen peroxide for 10 min to block endogenous peroxidase activity. Non-specific binding was blocked with 5% normal goat serum for 30 min at room temperature. Sections were incubated with primary antibodies against OPG or RANKL overnight at 4 °C, followed by incubation with secondary antibodies and visualization using DAB chromogen. Sections were counterstained with hematoxylin, dehydrated, cleared, and mounted. This procedure follows standard protocols (Table 1) [15]. Deparaffinized sections were stained in a Ventana XT automated staining system using 1:200 diluted primary antibodies (RANKL [sc-9073] and OPG [sc-11383], rat polyclonal IgG antibodies; Santa Cruz Biotechnology, Santa Cruz, CA, USA). The stained sections were examined under a light microscope (Olympus BX51, Tokyo, Japan). For immunohistochemical analysis, scoring was based on the intensity of staining. The intensity was classified as follows:

0: No staining.

+0.5: Very weak staining.

+1: Mild staining.

+2: Moderate staining.

+3: Strong staining [21].

**Figure 13:**
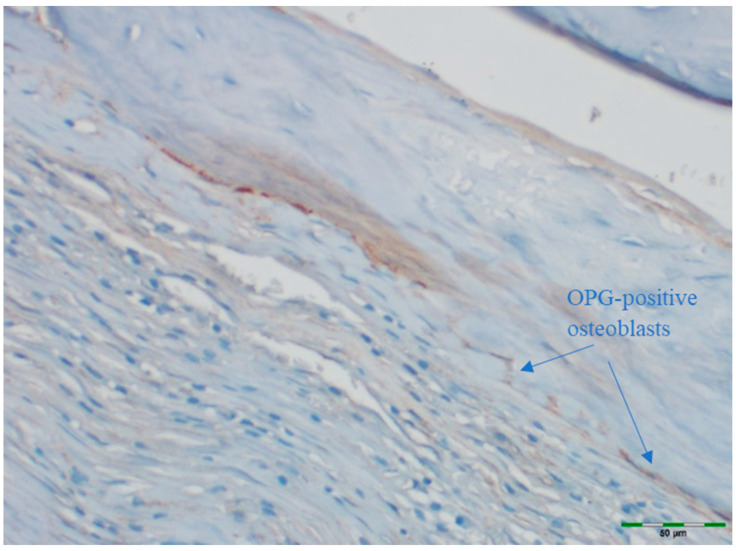
Immunohistochemical appearance of osteoblasts in the PRF group, showing moderate OPG staining (OPG immunostaining, 20× magnification). Arrows indicate OPG-positive osteoblasts (blue). Scale bar: 50 µm.

**Figure 14:**
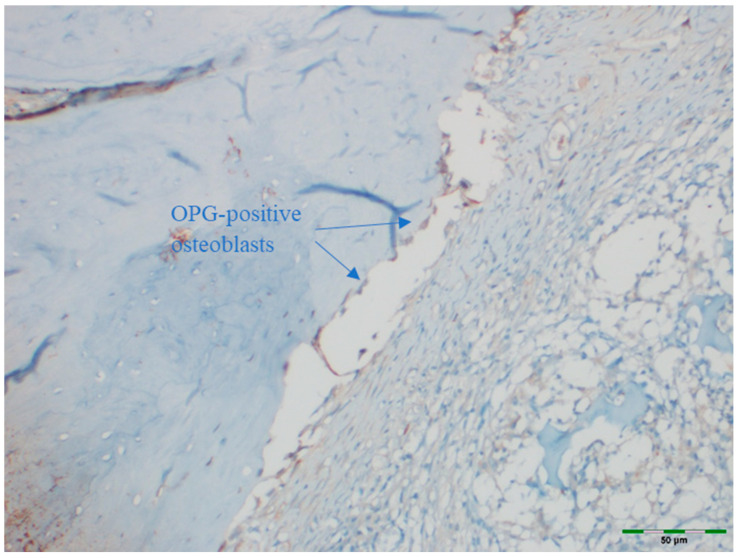
Immunohistochemical appearance of osteoblasts in the EMD group showing moderate OPG staining (OPG immunostaining, 20× magnification). Arrows indicate OPG-positive osteoblasts (blue). Scale bar: 50 µm.

**Figure 15:**
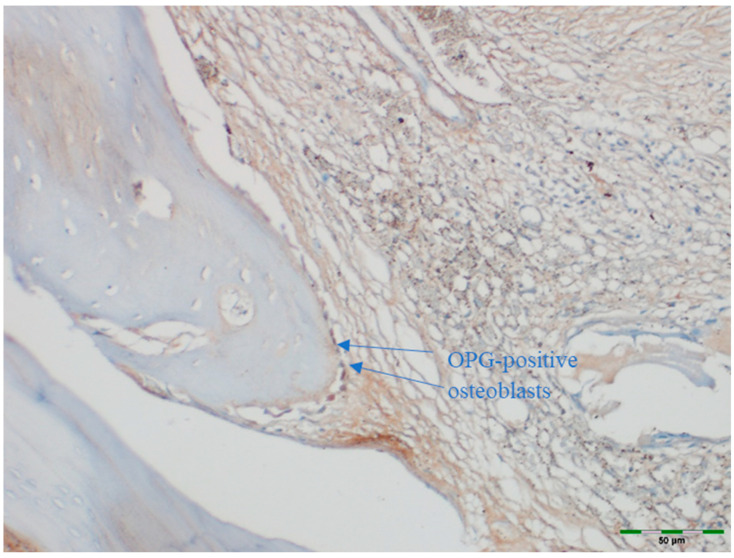
Immunohistochemical appearance of osteoblasts in the PRF + BG group showing strong OPG staining (OPG immunostaining, 20× magnification). Arrows indicate OPG-positive osteoblasts (blue). Scale bar: 50 µm.

**Figure 16:**
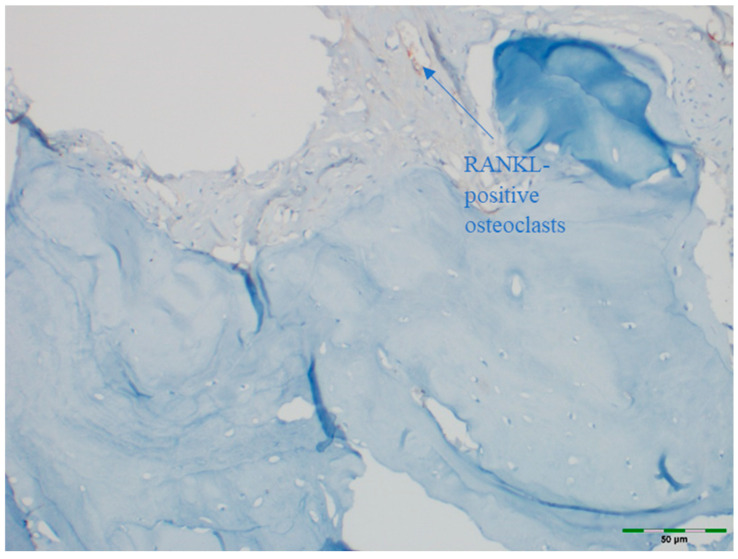
Immunohistochemical appearance of osteoclasts in the control group showing moderate RANKL staining (RANKL immunostaining, 40× magnification). Arrows indicate RANKL-positive osteoclasts (blue). Scale bar: 50 µm.

**Figure 17:**
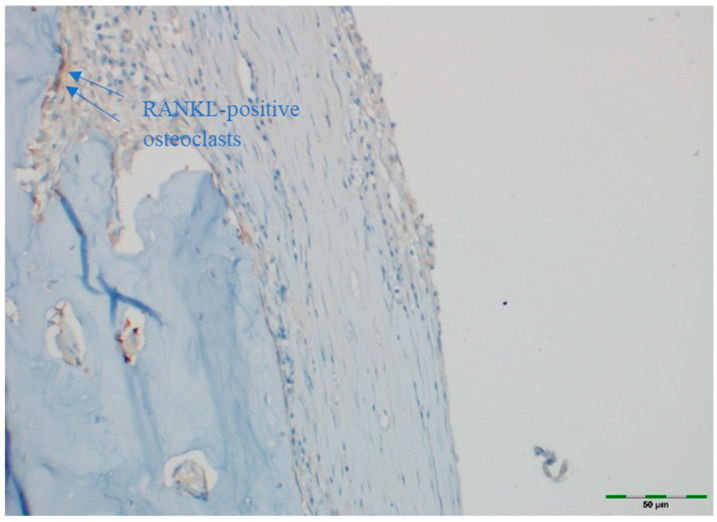
Immunohistochemical appearance of osteoclasts in the BG group showing intense RANKL staining (RANKL immunostaining, 40× magnification). Arrows indicate RANKL-positive osteoclasts (blue). Scale bar: 50 µm.

Images of all histological samples were captured using a digital camera attached to a light microscope at the original magnification and recorded on a computer. For histopathological analysis, an Olympus imaging system was used (Olympus BX51 light microscope; Olympus DP71 imaging system; Olympus, Tokyo, Japan) (Figure 13, Figure 14, Figure 15, Figure 16 and Figure 17). All histological and immunohistochemical evaluations were performed by an experienced pathologist who was blinded to the experimental group allocation. Tissue sections belonging to the same experimental group were examined in batches without any identifying labels, and group identities were disclosed only after completion of the scoring process [18].

### 2.6. Statistical Analysis

Statistical analyses were performed using SPSS Statistics (version 22; IBM Corp., Armonk, NY, USA). The study was not powered for factorial or interaction analyses due to limited group sizes (*n* = 13 per group). The normality of the data was assessed using the Kolmogorov–Smirnov and Shapiro–Wilk tests. The qualitative data were compared among groups using the chi-square and Fisher–Freeman–Halton tests. The levels of TNF receptor superfamily member 11b (TNFRSF11B/OPG) and TNF superfamily member 11 (TNFSF11/RANKL) were compared between groups using the Kruskal–Wallis test. A *p*-value of <0.05 was considered statistically significant.

## 3. Results

This study examined the effects of seven treatments (13 rats per treatment group). The various observations are described below.

### 3.1. New Bone Formation:

**Figure 18:**
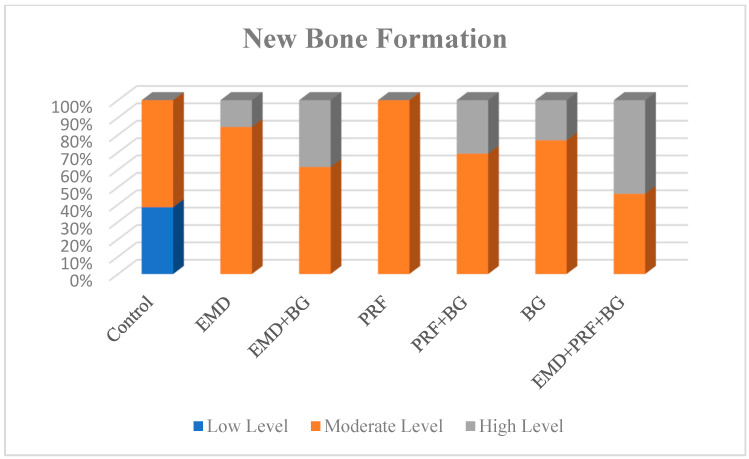
New bone formation scores in experimental groups. Moderate bone formation was highest in the PRF group compared with EMD + BG, PRF + BG and PRF + EMD + BG (*p* ≤ 0.05).

The amount of newly formed bone differed significantly among groups (*p*: 0.000; *p* < 0.05, Table 2). Post hoc pairwise analyses revealed that new bone formation was scored as mild significantly more often in the control group than in the EMD (*p* = 0.011), EMD + BG (*p* = 0.007), PRF (*p* = 0.047), PRF + BG (*p* = 0.024), BG (*p* = 0.007), and EMD + PRF + BG (*p* = 0.002) groups. In addition, new bone formation was scored as moderate bone significantly more often in the PRF group than in the EMD + BG (*p* = 0.039), PRF + BG (*p* = 0.030), and EMD + PRF + BG (*p* = 0.005) groups. Moreover, new bone formation was scored as moderate significantly more often in the EMD group than in the EMD + PRF + BG group (*p* = 0.039). However, the amounts of new bone levels did not differ significantly among the remaining groups (all *p* > 0.05) (Figure 18).

### 3.2. Osteoblast

The density of osteoblasts did not differ significantly among groups (*p* = 0.526). Osteoblast density was scored as dense in 38.5% of the control group, 53.8% of the EMD group, 61.5% of the EMD + BG group, 69.2% of the PRF group, 69.2% of the PRF + BG group, 76.9% of the BG group, and 69.2% of the EMD + PRF + BG group.

### 3.3. Osteoclasts

The density of osteoclasts did not differ significantly among groups (*p* = 0.570). The osteoclast density was scored as mild in 100% of the control group, 84.6% of the EMD group, 84.6% of the EMD + BG group, 84.6% of the PRF group, 92.3% of the PRF + BG group, 84.6% of the BG group, and 100% of the EMD + PRF + BG group.

### 3.4. Fibrosis

The extent of fibrosis did not differ significantly among groups (*p* = 0.081). Fibrosis was scored as moderate in 61.5% of the control group, 15.4% of the EMD group, 15.4% of the EMD + BG group, 46.2% of the PRF group, 38.5% of the PRF + BG group, 15.4% of the BG group, and 30.8% of the EMD + PRF + BG group.

### 3.5. Angiogenesis

The extent of angiogenesis did not differ significantly among groups (*p* = 0.567). The extent of angiogenesis was scored as moderate in 53.8% of the control group, 38.5% of the EMD group, 46.2% of the EMD + BG group, 53.8% of the PRF group, 30.8% of the PRF + BG group, 53.8% of the BG group, and 46.2% of the EMD + PRF + BG group.

### 3.6. OPG Levels

**Figure 19:**
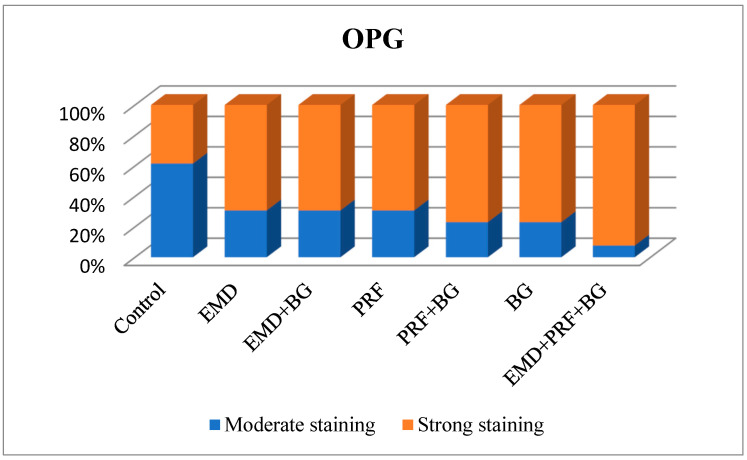
Osteoprotegerin (OPG) staining levels across groups. No significant differences were detected (*p* > 0.05).

OPG staining intensity did not differ significantly among groups (*p* = 0.147). OPG staining was scored as intense in 38.5% of the control group, 69.2% of the EMD group, 69.2% of the EMD + BG group, 69.2% of the PRF group, 76.9% of the PRF + BG group, 76.9% of the BG group, and 92.3% of the EMD + PRF + BG group. Although the difference was not statistically significant, OPG staining intensity was weakest in the control group and strongest in the EMD + PRF + BG group (Figure 19).

### 3.7. RANKL Expression

**Figure 20:**
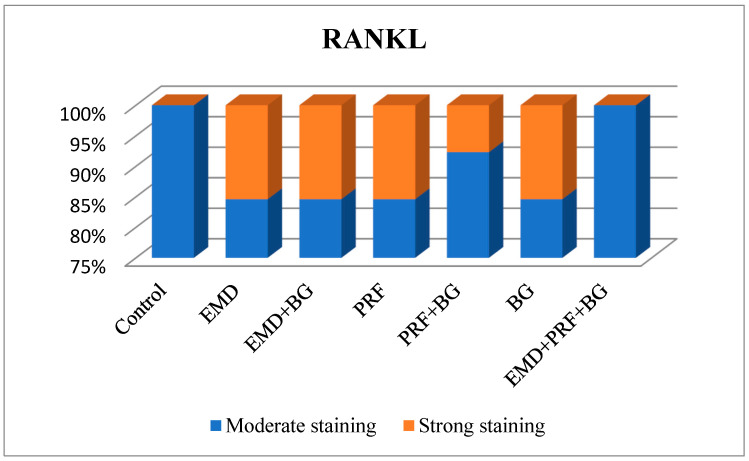
Receptor activator of nuclear factor-κB ligand (RANKL) staining levels across groups, showing no significant intergroup variation (*p* > 0.05).

RANKL staining intensity did not differ significantly among groups (*p* = 0.570). RANKL staining intensity was scored as moderate in 100% of the control group, 84.6% of the EMD group, 84.6% of the EMD + BG group, 84.6% of the PRF group, 92.3% of the PRF + BG group, 84.6% of the BG group, and 100% of the EMD + PRF + BG group (Figure 20).

### 3.8. OPG/RANKL Ratio

The OPG/RANKL ratio did not differ significantly among groups (*p* > 0.05).

## 4. Discussion

The biological principle of GTR was developed in the context of tissue regeneration. According to this principle, regeneration of a specific tissue type is achieved when cells capable of replacing the lost tissue populate the defect site during the healing process [22]. In GTR, both natural and synthetic biomaterials with osteogenic potential are utilized. The choice of graft biomaterial plays a crucial role in modulating wound healing from repair to regeneration. Signaling molecules present within autografts and allografts can stimulate regeneration. Therefore, biomaterials such as EMD, PRF, PRP, bone grafts, growth factors, BMPs, and resorbable or non-resorbable membranes are often employed in GTR [23].

Previous studies have reported the stages of bone healing evaluated weekly. Lundgren et al. demonstrated that after cortical decortication of the parietal bone in rabbits, the newly formed bone tissue following a three-month healing period consisted of thin bony trabeculae dispersed within abundant marrow spaces [24]. Bodde et al. defined the healing period for critical-sized bone defects as 12 weeks [25]. Therefore, a three-month healing period was selected in our study for the histopathological and immunohistochemical evaluation and comparison of newly formed bone following GTR.

Scoring methods provide quantitative measures for histopathological evaluation. Toker et al. and Sacak et al. developed a semi-quantitative scoring system to assess new bone formation (0 = no bone formation, 1 = slight visible bone formation, 2 = moderate visible bone formation, and 3 = high level of visible bone formation) [26,27]. In our study, H&E-stained histological sections were evaluated semi-quantitatively for various parameters, including osteoblast and osteoclast density, new bone formation, fibrosis, and angiogenesis, using this scoring system.

Franke Stenport and Johansson conducted a six-week study in which EMD was applied around 36 titanium implants inserted into rabbit femurs, reporting no significant difference in new bone formation [28]. Kawana et al. demonstrated that EMD has an osteopromotive effect supporting bone regeneration during the healing of long bones [29]. Similarly, Yoneda et al. reported that applying EMD accelerated new bone formation in a rat calvarial defect model [30]. Consistent with these studies, our results showed that new bone formation did not differ significantly between the EMD and control groups. However, EMD increased osteoblastic and osteoclastic activity and reduced fibrosis. Therefore, EMD can support bone formation by stimulating the cells and signaling molecules involved in bone regeneration, thereby accelerating the healing process.

The effect of EMD combined with bone xenograft on bone formation has been investigated around implants placed in dog mandibles by Casati et al. and in cylindrical defects created in rat femurs by Donos et al. Both studies observed an increase in new bone formation in these models; however, the differences were not statistically significant [31,32]. Similarly, in our study, the rate of advanced bone formation, as assessed histopathologically, was greater in the EMD + BG group than in the control and BG groups; however, this difference was not statistically significant. It is considered that the osteoconductive properties of the bone graft and the accelerated healing effect of the EMD act synergistically to enhance bone formation when used together.

Kang et al. reported that PRF is a potent factor in sustaining tissue regeneration. Their eight-week study using rat calvarial bones showed favorable results in terms of new bone formation in the PRF-treated group compared to the control group; however, the difference was not statistically significant [33]. Similarly, Li et al. demonstrated that PRF alone promoted mineralization of alveolar bone, indicating that fibrin plays a vital role in osteogenic differentiation [34]. Consistent with these studies, in our study, the PRF group exhibited a higher rate of moderate new bone formation than the control group; however, this difference did not reach statistical significance. Nonetheless, using PRF alone supports new bone formation by enhancing vascularization and promoting osteogenic cell differentiation, suggesting that it is an effective material for GTR.

Oliveira et al. conducted a study involving 48 rats to examine the interaction between PRF and bone xenografts in rat calvaria. At 60 days postoperatively, PRF demonstrated a synergistic effect with bone xenograft during bone regeneration. Although the PRF-xenograft combination was superior to xenograft alone, the difference was not statistically significant [35]. Choukroun et al. documented that while PRF does not increase cellular proliferation in the long term, it plays a significant role in graft revascularization by supporting angiogenesis [36]. In our study, histopathological examination revealed a higher rate of advanced new bone formation in the PRF + BG group than in the BG group; however, this difference was not statistically significant. These findings are consistent with those of similar studies. We propose that when used in combination with bone grafts, PRF can positively influence angiogenesis and accelerate early osteogenic activity, potentially resulting in more favorable and faster bone formation compared to bone grafts alone.

In a study by Qu et al., adding EMD to the medium in which osteoblasts were cultured on a titanium surface resulted in a significant increase in alkaline phosphatase activity and bone gamma-carboxyglutamate protein (BGLAP, formerly osteocalcin [OCN]) production, as well as elevated OPG levels but not RANKL levels. Interestingly, it also reduced cell viability and proliferation in the extracellular environment [37]. In our study, although the densities of osteoblasts and osteoclasts increased in the groups treated with EMD, the differences were not statistically significant, unlike the findings of Qu et al. This discrepancy may be due to differences in experimental setups: Qu et al. conducted an in vitro monoculture study with a single cell type, whereas our study applied EMD to living tissues in vivo, which is a more complex microenvironment.

Sumida et al. evaluated the role of PRF in bone formation using a bone defect model with cultured osteoblasts. They found that PRF increased OPG production in the cultured bone cells over 12 days but did not affect RANKL production, thereby increasing the OPG/RANKL ratio. OPG expression was higher in the PRF-treated group than in the untreated control group. Therefore, their findings suggest that PRF enhances osteoblast differentiation by increasing OPG production and the OPG/RANKL ratio, thereby promoting early-stage osteogenesis [38]. In our study, OPG levels and the OPG/RANKL ratio did not differ significantly among groups, which is inconsistent with the findings of Sumida et al. This discrepancy may be explained by the longer duration of our study (three months), during which OPG may have been predominantly expressed during the earlier healing phases. Nonetheless, in our study, RANKL levels did not differ significantly among groups, consistent with the findings of Sumida et al. Thus, consistent with Sumida et al., our findings indicate that PRF does not significantly affect RANKL levels but may exert a more pronounced effect on OPG activity during the early stages of healing. Although materials such as PRF may not influence bone formation as strongly as barrier membranes in GTR, their use is considered effective in enhancing angiogenesis and supporting bone healing.

Gupta et al. compared bone formation in intraosseous defects treated with EMD and PRF without bone grafts. They reported that both materials were equally effective in improving clinical attachment level and reducing pocket depth, but EMD demonstrated superior bone formation in intraosseous defects [39]. In our study, new bone formation did not differ significantly between the PRF-treated groups (which showed moderate bone formation) and the EMD group, consistent with the findings of Gupta et al. Histopathologically, the rate of high-level bone formation was greater in the EMD group than in the PRF group. This finding may be explained by EMD’s superior role in enhancing angiogenesis, promoting the release of growth factors, and stimulating osteoblasts for bone formation compared to PRF.

In addition, the findings of Türkal et al. provide clinical support for our observations. In their randomized clinical trial on intrabony periodontal defects, both EMD and the combination of EMD with PRF resulted in significant improvements in probing depth and clinical attachment level, although no significant intergroup differences were reported [40]. Consistent with these results, our experimental data indicate that while the combined application of EMD, PRF, and bone graft shows beneficial effects on bone regeneration, the extent of their synergistic interaction remains limited, suggesting that their main advantage lies in providing comparable regenerative outcomes across different defect models.

Although this study utilized a rat calvarial defect model, the findings have potential translational relevance for human clinical applications. EMD, PRF, and bone grafts are widely used in dental and craniofacial regenerative procedures, and our comparative preclinical data provide insight into the relative efficacy of these treatments. However, differences in bone healing rates, defect size, and physiology between rats and humans should be considered when extrapolating these results. Future studies using larger animal models or clinical trials are warranted to confirm these translational implications.

## 5. Limitations

Our study had several limitations. Firstly, new bone formation was only assessed based on histopathological and immunohistochemical parameters; therefore, future studies should also evaluate bone formation volumetrically using imaging techniques, such as micro-computed tomography. Secondly, the histopathological scoring system used is subjective and might not fully capture all details; therefore, future studies should also consider additional approaches. Finally, treatment effects were only evaluated at a single time point; therefore, future studies should evaluate the effects at additional time points, such as after 7, 14, and 21 days, to better understand early cellular events. Additional angiogenic or osteogenic markers were not included; future studies will expand the molecular panel. Finally, the study was not designed to evaluate synergistic or redundant interactions between EMD, PRF, and bone grafts; future studies with larger cohorts are warranted to investigate these effects.

## 6. Conclusions

Within the limitations of this study, the use of EMD and PRF, alone or in combination with bone grafts, appears to promote new bone formation and influence bone cell activity in guided tissue regeneration (GTR). These materials play a significant role in treating periodontal defects, peri-implantitis, implant surgeries, pre-implant bone augmentation, and bone defects caused by trauma or malignancy. This study provides comparative preclinical evidence on the effects of Emdogain (EMD), platelet-rich fibrin (PRF), and bone grafts, alone or in combination, on bone formation in rat calvarial defects. By assessing multiple biomaterial combinations, our findings contribute to understanding the relative and potential synergistic effects of these regenerative materials, which may inform the design of future preclinical and clinical studies in GTR.

## Figures and Tables

**Table 1:** Overview of the histological and immunohistochemical procedures used in this study, including main steps with duration and temperature.

Step	Procedure	Description	Duration	Temperature
1	Fixation	Samples fixed in 10% formalin solution	72 h	Room temp
2	Decalcification	Immersion in 10% formic acid; solution changed every 2 days	1 week	Room temp
3	Washing	Running tap water to remove residual acid	12 h	Room temp
4	Dehydration	Immersion in graded ethanol series (70%, 80%, 95%, 100%)	~2 h total	Room temp
5	Clearing	Immersion in xylene	30 min	Room temp
6	Paraffin embedding	Infiltration with melted paraffin wax	Overnight	60 °C
7	Sectioning	Paraffin blocks cut into 5 µm sections	–	Room temp
8	Deparaffinization	Sections incubated at 65 °C, then immersed in xylene and alcohol	1 h + washes	65 °C and RT
9	Rehydration	Stepwise rehydration with graded ethanol to distilled water	~20 min	Room temp
10	H&E staining	Hematoxylin and eosin staining for histology	Standard Protocol	Room temp
11	Antigen retrieval	Heat-induced epitope retrieval for IHC	20 min	95–98 °C
12	Blocking	Non-specific binding blocked with serum	30 min	Room temp
13	Immunohistochemistry	OPG and RANKL primary antibody incubation, followed by secondary antibody and DAB visualization	Overnight (primary), 1 h (secondary)	4 °C (primary), RT (secondary)

**Table 2:** Evaluation of the groups based on the levels of new bone formation.

New Bone Formation	Control	EMD	EMD + BG	PRF	PRF + BG	BG	EMD + PRF + BG	
	(*n* = 13)	(*n* = 13)	(*n* = 13)	(*n* = 13)	(*n* = 13)	(*n* = 13)	(*n* = 13)	
	*n* (%)	*n* (%)	*n* (%)	*n* (%)	*n* (%)	*n* (%)	*n* (%)	*p*
Low Level	5	0	0	0	0	0	0	0.000 *
	(38.5%)	(0%)	(0%)	(0%)	(0%)	(0%)	(0%)	
Moderate Level	8	11	8	13	9	10	6	
	(61.5%)	(84.6%)	(61.5%)	(100%)	(69.2%)	(76.9%)	(46.2%)	
High Level	0	2	5	0	4	3	7	
	(0%)	(15.4%)	(30.8%)	(0%)	(30.8%)	(23.1%)	(53.8%)	

Chi-square test * *p* < 0.05.

## Data Availability

The data that support the findings of this study are available from the corresponding author upon reasonable request.

## Abbreviations

Abbreviation	Full Term
GTR	Guided Tissue Regeneration
BMPs	Bone Morphogenetic Proteins
EMD	Enamel Matrix Derivative
BG	Bone Graft
PRF	Platelet-Rich Fibrin
ENAM	Enamelin
AMBN	Ameloblastin
AMTN	Amelotin
ODAM	Odontogenic Ameloblast-Associated Protein
TGF-β	Transforming Growth Factor-beta
PRP	Platelet-Rich Plasma
OPG	Osteoprotegerin
RANKL	Receptor Activator of Nuclear Factor Kappa-B Ligand
H&E	Hematoxylin and Eosin
TNF	Tumor Necrosis Factor
DAB	3,3′-Diaminobenzidine
BGLAP	Bone Gamma-Carboxyglutamic Acid-Containing Protein
OCN	Osteocalcin
